# Systematic Analysis of FASTK Gene Family Alterations in Cancer

**DOI:** 10.3390/ijms222111337

**Published:** 2021-10-20

**Authors:** Lorena Magraner-Pardo, Dino Gobelli, Miguel A. de la Fuente, Tirso Pons, María Simarro

**Affiliations:** 1 Prostate Cancer Clinical Research Unit, Spanish National Cancer Research Center (CNIO), 28029 Madrid, Spain; lorena.magraner@gmail.com; 2 Department of Cell Biology, Histology and Pharmacology, University of Valladolid, 47005 Valladolid, Spain; djgobelli@gmail.com (D.G.); miguelafuente@gmail.com (M.A.d.l.F.); 3 Unit of Excellence, Institute of Biology and Molecular Genetics, University of Valladolid and CSIC, 47003 Valladolid, Spain; 4 Department of Immunology and Oncology, National Center for Biotechnology (CNB-CSIC), Spanish National Research Council, 28049 Madrid, Spain; tirsopons@gmail.com; 5 Department of Department of Nursing, University of Valladolid, 47005 Valladolid, Spain

**Keywords:** mitochondria, FASTK, cancer, genetic alterations

## Abstract

The FASTK family of proteins have been recently reported to play a key role in the post-transcriptional regulation of mitochondrial gene expression, including mRNA stability and translation. Accumulated studies have provided evidence that the expression of some FASTK genes is altered in certain types of cancer, in agreement with the central role of mitochondria in cancer development. Here, we obtained a pan-cancer overview of the genomic and transcriptomic alterations of FASTK genes. FASTK, FASTKD1, FASTKD3 and FASTKD5 showed the highest rates of genetic alterations. FASTK and FASTKD3 alterations consisted mainly of amplifications that were seen in more than 8% of ovarian and lung cancers, respectively. FASTKD1 and FASTKD5 were the most frequently mutated FASTK genes, and the mutations were identified in 5–7% of uterine cancers, as well as in 4% of melanomas. Our results also showed that the mRNA levels of all FASTK members were strongly upregulated in esophageal, stomach, liver and lung cancers. Finally, the protein-protein interaction network for FASTK proteins uncovers the interaction of FASTK, FASTKD2, FASTKD4 and FASTKD5 with cancer signaling pathways. These results serve as a starting point for future research into the potential of the FASTK family members as diagnostic and therapeutic targets for certain types of cancer.

## 1. Introduction

The Fas-activated serine/threonine kinase (FASTK) family of proteins has recently emerged as a key post-transcriptional regulators of mitochondrial gene expression. It contains six members: FASTK, the founding member, and its homologs FAST Kinase Domains 1–5 (FASTKD1–5) [1]. The six proteins have been found only in vertebrates and share three homology domains called FAST_1, FAST_2, and RAP [1]. According to homology predictions, the RAP (an acronym for RNA-binding domain abundant in apicomplexans) domain is a putative RNA-binding domain [2], whereas the functions of the FAST_1 and FAST_2 domains remain unknown. Despite being architecturally related, each member has a different function in the regulation of mitochondrial RNA biology, from mRNA processing and maturation to ribosome assembly and translation. Consequently, the deletion of some of these genes results in altered composition and activity of various OXPHOS complexes. We have recently reviewed elsewhere the information available on the molecular mechanisms of action of each of the FASTK proteins [3].

Little is known about the link between this family of proteins and disease. FASTKD2 is the first and so far only member of the family that has been found to be mutated in a mitochondrial disease. Ghezzi et al. found homozygosity for a nonsense mutation in FASTKD2 in 2 siblings with mitochondrial encephalomyopathy associated with cytochrome c oxidase deficiency [4]. Recently, a second report has identified FASTKD2 compound heterozygous mutations in a patient with adult-onset MELAS-like syndrome [5]. From an immunological aspect, our previous work has revealed the importance of FASTK in regulating inflammation and the phagocytosis of bacteria by macrophages in various disease models using FASTK knockout mice [6,7,8].

In addition to the abovementioned reports, there is a remarkable abundance of microarray-based gene expression studies that reveal high expression levels of certain FASTK family genes in various types of cancer. FASTK showed strong overexpression in highly malignant pancreatic ductal adenocarcinoma and in the various types of cystic pancreatic tumors with potential for malignant transformation [9]. It has also been found to be overexpressed in mycosis fungoides, the most common cutaneous T-cell lymphoma. In the case of mycosis fungoides, overexpression of FASTK was associated with a chromosomal alteration consisting of a gain of 7q36, where the FASTK gene resides [10]. Another study identifies FASTK as a direct target of miR-106a-5p, a microRNA whose downregulation in astrocytomas is associated with poor prognosis [11,12]. Accordingly, FASTK was found to be upregulated and positively associated with advanced clinical stages of astrocytoma [12]. Moreover, the same study shows that both the expression of miR-106a-5p and the knockdown of FASTK on astrocytoma cells inhibit cell proliferation and migration and can promote cell apoptosis [12].

Another member of the FASTK gene family, FASTKD1, has been identified as highly sensitive RNA-based biomarker for endometrial carcinoma in uterine aspirate samples [13]. Its overexpression has also been identified as a poor prognostic factor in child and adult patients with acute lymphoblastic leukemia (ALL) through meta-analysis on publicly available gene expression datasets [14]. The prognostic value of FASTKD1 overexpression was further validated in a prospective cohort of 62 ALL adult patients [14]. Like FASTK, FASTKD2 was upregulated in pancreatic cancer tissues and was associated with poor prognosis [15]. In contrast, high expression of FASTKD3 was associated with increased survival in bladder cancer [16]. To date, there are no studies that describe altered expression of FASTKD4 or FASTKD5 in cancer.

Finally, we highlight a few in vitro studies that provide mechanistic links between FASTK, FASTKD2 and cancer. First, two studies by the same research group show that FASTKD2 is highly proapoptotic in various cancer cell lines and demonstrate that its expression is tightly controlled by the transcriptional complex NRIF3-DIF-1 in breast and prostate cancer cell lines [17,18]. It should be noted that FASTKD2 was identified as the only member of the FASTK family with proapoptotic activity [18]. For all the above, it is surprising that FASTKD2 has recently been reported to promote the growth and invasion of pancreatic cancer cells in a c-Myc-dependent manner [15]. With regard to the cancer signaling pathways in which FASTK is involved, it has been described that the efficient splicing of FASTK pre-mRNA requires PHF5A, a key splicing factor in tumor progression [19]. PHF5A ablation enhances apoptosis in a breast cancer cell line and further promotes the production of a short FASTK variant that retains intron 5, which leads to the generation of a premature stop codon. This newly identified FASTK splice variant has proapoptotic activity and was proposed to be responsible, at least in part, for the increase in apoptosis when PHF5A is ablated [19].

In this study, we investigated the genomic and transcriptomic alterations for all FASTK genes across 33 different human cancer types using data generated by The Cancer Genome Atlas (TCGA) and constructed the protein-protein interaction network for FASTK proteins.

## 2. Results

### 2.1. Genetic Alterations of FASTK Family Genes across Human Cancers

Cancer is a disease caused by the accumulation of genetic alterations. On average, cancer samples contain 4–5 driver alterations the majority of which are mutations or deletions of tumor suppressor genes (i.e., TP53, BRCA2, APC), and amplifications of oncogenes (i.e., MYC, MCL1, CCND1) [20,21]. The genetic alterations show extensive variation across cancer types and in around 5% of samples no drivers are identified suggesting that cancer driver discovery is not yet complete [20]. As summarized in the introduction section, several works have revealed the prognostic value of FASTK genes expression in various cancers; however, until now, no genetic alterations of these genes have been described across cancers. In order to systematically identify genetic alterations within FASTK genes, we analyzed sequencing data of 33 cancer types in TCGA by using the online bioinformatics tools cBioPortal and IntOGen (see Materials and Methods). The number of samples and the 2–4 letter codes for each cancer type are listed in Table 1.

**Figure 1:**
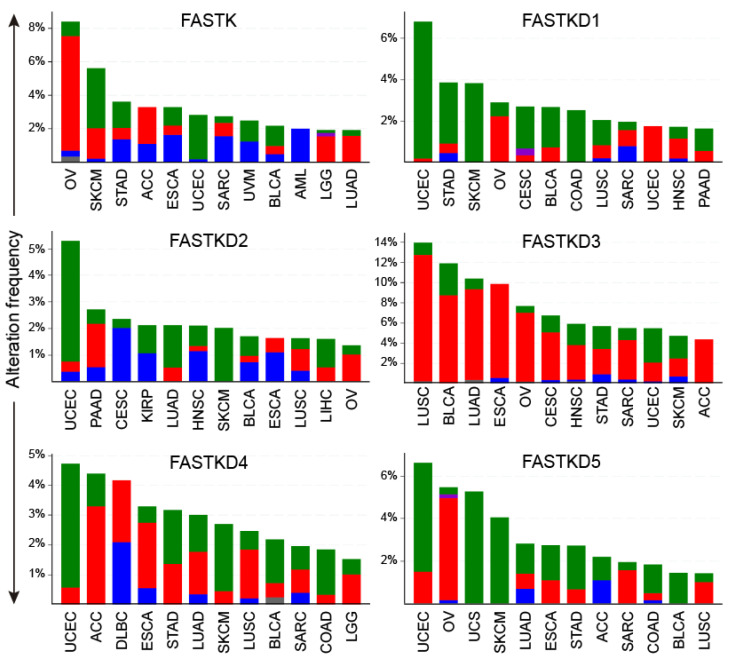
Alterations of the FASTK gene family in cancer. Distribution of the genomic alterations of each member of the FASTK family across the 12 tumor types harboring the highest alteration rates. Frequencies in tumors not shown are provided in Table S2. Colors represent different types of alterations: red-amplification, green-mutation, blue-deletion, purple-fusion and grey-multiple alterations. Figures were generated with the cBioPortal online tool.

The frequencies of genetic alterations for each of the FASTK genes across the different types of cancer are provided in Figure 1 and Table S1. We highlight here the most noteworthy changes. FASTK gene alterations were most frequent in OV (>8%) and consisted mainly of amplifications. Mutational alterations of FASTK were detected at a frequency of nearly 4% in SKCM. FASTKD1, FASTKD2, FASTKD4 and FASTKD5 exhibited the highest mutation frequencies in UCEC and SKCM. Among them, FASTKD1 and FASTKD5 were the most frequently mutated in both UCEC (6.6% and 5.1%, respectively) and SKCM (3.8% and 4%, respectively). UCEC and SKM are two of the tumors with the highest mutational burdens [22]. We tested whether FASTKD1-mutated samples and FASTKD5-mutated samples showed higher mutation rates in other genes compared to samples with unaltered FASTKD1 and FASTKD5 genes. As expected, we observed a significant co-occurrence of mutated FASTKD1 and mutated FASTKD5 with mutations in other genes, including several of the ten most frequently mutated genes in UCEC and SKCM. Importantly, in the list of top-ranked mutated genes we found TTN (coding for Titin: 34,350 amino acids) and MUC16 (coding for Mucin-16: 14,507 amino acids). Both genes have a high risk of residue alterations because of random DNA repair errors. Therefore, most missense mutations in these genes are likely to be ‘passenger’ mutations, and their role in cancer progression remains to be evaluated [23]. On the contrary, other genes in the list (i.e., PIK3CA, PTEN, BRAF) are well-known tumor suppressor genes or oncogenes in many cancers [23]. The co-occurrence between mutated FASTK genes and mutations in other genes in the UCEC samples was more statistically significant than that in the SKCM samples (Figure S1).

On the other hand, the predominant FASTKD3 genetic alterations consisted of amplifications that were found in 12.5% of LUSC, 9% of LUAD, 9.3% of ESCA and 8.7% of BLCA. We analyzed the correlation between FASTKD3 expression and copy number alterations (CNAs) in those tumors. As shown in Figure S2, CNAs were significantly correlated with FASTKD3 expression. Finally, we show in Figure S3 the cumulative frequencies of the above-described genetic alterations of FASTK genes across the different types of cancer.

### 2.2. Recurrent Mutations in FASTK Genes

**Figure 2:**
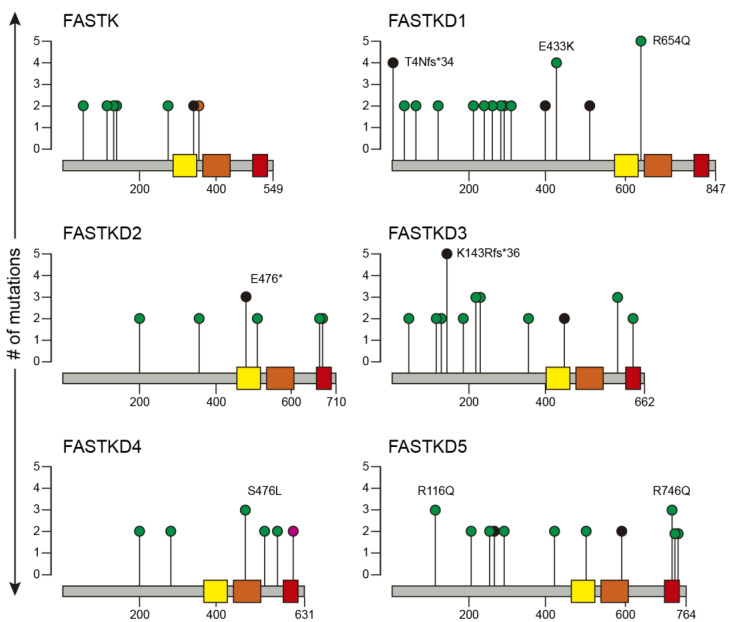
Lolliplots showing identified variants relative to a schematic representation of the protein (figure adapted from the lolliplots downloaded from cBioPortal). *X*-axis shows the amino acid position and *y*-axis the number of mutations. Positions which are recurrently mutated (total count ≥ 3) are labelled with text specifying the amino acid changes. Colors represent different types of mutations: green-missense, black-truncating, orange-splicing and magenta-silent. FAST_1 FAST kinase-like domain 1(yellow), FAST_2 FAST kinase-like domain 2 (orange), RAP RNA binding domain abundant in apicomplexans (red). A complete description of the mutations can be found in Table S2.

It has been widely reported that genes that are mutated in only a small fraction (<1%) of tumors can still act as drivers [24]. Therefore, even though we had found that mutations in FASTK genes are rarely found in cancer, we decided to explore their distribution across multiple cancer types. Figure 2 illustrates mutations in FASTK genes recurring in more than two tumor samples. FASTKD1, FASTKD3 and FASTKD5 were the FASTK genes most frequently mutated in cancers.

The most recurrent mutations in FASTKD1 were frameshift mutation T4Nfs*34 and missense mutations E433K and R654Q and in FASTKD3, the frameshift mutation K143Rfs*36 and missense mutations R207C, R216C and R590M. Finally, the most frequent mutations in FASTKD5 were missense mutations R116Q and R746Q. It is worth noting that most mutations in all FASTK genes were positioned outside the C-terminal shared domains across FASTK proteins: FAST_1, FAST_2 and RAP. A complete description of the FASTK genes mutations and the types of cancer where they were found are available in Table S2.

### 2.3. Expression Profile of FASTK Genes across Human Cancers

As reviewed in the introduction, several studies have previously reported upregulation of FASTK, FASTKD1, FASTKD2 and FASTKD3 in certain types of cancer. Here, we took advantage of RNA-sequencing datasets available at the TCGA Data Portal and the UALCAN online tool to expand our knowledge on the expression of all the FASTK members across cancers. For the analysis of differential expression, we only considered tumor types for which we had ≥10 matched normal samples. The dataset contained 6903 and 709 samples for 16 cancer types and matched normal tissues, respectively (Table S3).

**Figure 3:**
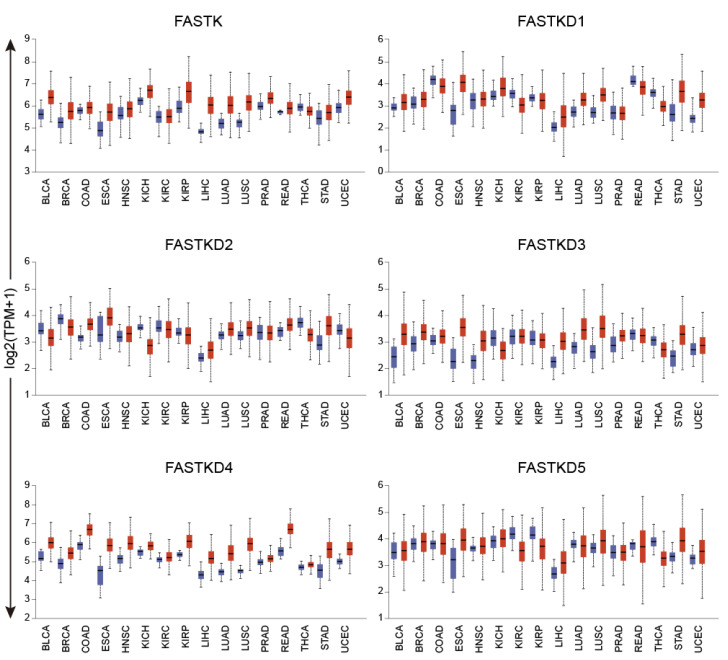
Expression of FASTK genes across human cancers. Boxplots showing the expression of all FASTK genes across 16 cancer types (red) and matched normal tissues (blue). The X-axes represent the name of each type of cancer, while the Y-axes represent the gene expression values as log2 (TMP + 1). *p* values are provided in Table S3. Figures were generated with the UALCAN online tool.

As shown in Figure 3, the expression of the FASTK genes was altered across several cancer types. The most remarkable change was a strong upregulation of all FASTK genes in two types of gastrointestinal cancers, ESCA and STAD, and also in LIHC and LUSC.

In certain tumors, however, the different FASTK genes showed different and even opposite expression patterns. For example, in BRCA and BLCA, FASTK, FASTKD3 and FASTKD4 were significantly upregulated while FASTKD2 was downregulated. In the case of THCA, all FASTK genes were downregulated except FASTKD4 which was mildly upregulated. The statistical significance of these expression changes is provided in Table S3. As shown in Figure S4, these results were confirmed using the GEPIA2 tool (see Materials and Methods).

### 2.4. Protein Interaction Network of FASTK Members: Pathways and Molecular Functions Enrichment Analysis

**Figure 4:**
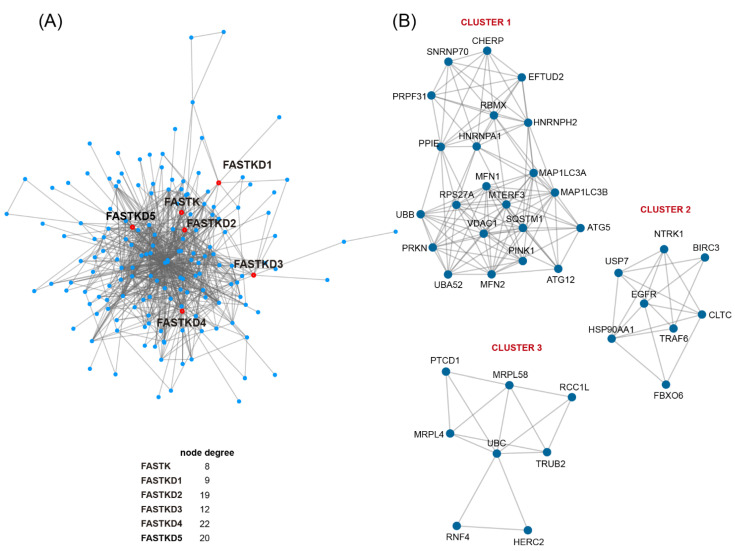
Protein interaction network of FASTK members. Left panel (**A**) shows the FASTKs PPI network. Right panel (**B**) shows the three clusters of most densely connected proteins and the Gene Set/Pathway enrichment analyses.

We next performed protein-protein interaction (PPI) network analysis to explore the possible relationship between FASTK genes and cancer signaling pathways. We obtained a FASTKs PPI network consisting of 185 nodes and 278 edges. We next identified three clusters of highly connected proteins and conducted pathway and gene set enrichment analyses (Figure 4).

As shown in Table 2, cluster 1-related functions were mitophagy and selective autophagy, pathways in cancer and endocytosis for cluster 2, and mitochondrial translation and gene expression and processing of DNA double-strand break ends for cluster 3.

FASTK proteins connections with the clusters are as follows: FASTK interacts with HNRNPH2 (cluster 1), HSP90AA1 (cluster 2), and UBC (cluster 3); FASTKD1 interacts with MRPL58 and UBC (cluster 3); FASTKD2 interacts with UBC, MRPL4 and MRPL58 (cluster 3), CLTC and USP7 (cluster 2), RBMX, TRAF6, and HNRNPA1 (cluster 1); FASTKD3 interacts with PTCD1 (cluster 3); TBRG4 interacts with EGFR and NTRK1 (cluster 2) and UBC (cluster3); and FASTKD5 interacts with NTRK1 and FBXO6 (cluster 2) and HERC2 and UBC (cluster 3). These interesting results not only reveal the known relationship of FASTK proteins to mitochondrial physiology, but also uncover the particular interaction of FASTK, FASTKD2, FASTKD4 and FASTKD5 with cancer signaling pathways.

## 3. Discussion

In this study, we provide a novel systematic analysis of FASTK gene family alterations in cancer. FASTK, the founding member and its homologs FASTKD1–5 are architecturally related RNA-binding proteins, each having a different function in the regulation of mitochondrial RNA biology, from mRNA processing and maturation to ribosome assembly and translation. Accordingly, the silencing or mutation of these genes leads to different alterations in the assembly and activity of various OXPHOS complexes containing mitochondrially encoded subunits [3]. For example, FASTK is required for MT-ND6 mRNA stabilization and thus for complex I activity, FASTKD2 for mitochondrial translation and processing and expression of MT-ND6 mRNA, and FASTKD3 for MT-CO1 mRNA translation and complex IV activity. Finally, FASTKD4 and FASTKD5 are necessary for maturation of mRNAs that cannot be processed by the activities of RNase P and RNase Z: the non-canonical MT-ND5 + MT-CYB RNA precursor in the case of FASTKD4, and non-canonical MT-ATP8/6 + MT-CO3, MT-ND5 + MT-CYB and ncRNA + MT-CO1 precursor transcripts [3].

It has been widely reported that cancer cells exhibit different metabolic phenotypes with variable participation of both OXPHOS and glycolysis. Interestingly, metastatic cells utilize OXPHOS as a preferred metabolic pathway for ATP generation. This dependence converts OXPHOS in a promising target in antimetastatic therapy [25]. Of note, several clinical trials of drugs targeting OXPHOS complexes I-IV are currently underway [25]. As reported in the results section, one remarkable finding was the upregulation of all FASTK genes in various types of cancer: ESCA, STAD, LIHC and LUSC. Our analysis also confirmed the upregulation of FASTKD1 in UCEC reported by others [13]. However, we were unable to confirm some of the previously reported increases in the expression of FASTK genes when the tumor was not included in the TCGA database or had less than 10 matched normal samples. The upregulation of FASTK genes in cancer tissues could potentially contribute to the OXPHOS overload in metastatic cancer cells. It will be interesting to explore how silencing FASTK genes will impact the proliferative and migratory capacity of cell lines derived from these types of cancers, as a first step to explore their potential as therapeutic targets.

In this study, our analysis of the TCGA sequencing data revealed that FASTK and especially FASTKD3 amplifications stand out as the most common genetic alterations of FASTK genes across 33 cancer types. Of note, FASTKD3 was found to be amplified in >8% of various types of cancer including LUSC, LUAD, ESCA and BLCA. Gene amplification is a typical genetic alteration in cancer, and many oncogenes have been identified in the amplified regions. In this regard, novel cancer-associated genes might remain to be identified in the amplified regions [26] and, among these genes, FASTK and FASTKD3 could contribute to tumorigenesis in concert with oncogenes in the same amplicons. Here, we also provide a complete description of the FASTK genes mutations and the types of cancer where they were found. FASTK genes are rarely mutated in tumor samples and we applied the IntOGen online tool (see Materials and Methods), which did not identify cancer driver mutations in FASTK genes. However, this does not rule out the possibility that FASTK genes mutations may modulate the oncogenic potential of driver mutations. FASTKD1, FASTKD3 and FASTKD5 were the most commonly mutated FASTK genes and most mutations were outside the C-terminal shared domains FAST_1, FAST_2 and RAP. The region of the FASTK family members between the mitochondrial targeting sequence and the conserved C-terminal domains do not contain any recognizable domains with known function. It will be interesting to characterize the molecular and functional consequences of these mutations in parallel with the upcoming discoveries about the functions of the different parts of the FASTK proteins.

Finally, we provide novel evidence of the PPI network of FASTK proteins, which uncovers the interaction of FASTK, FASTKD2, FASTKD4 and FASTKD5 with cancer signaling pathways. Among the FASTKs interactors involved in cancer development, the best understood ones are EGFR, NTRK1 and FBXO6. Strikingly, EGFR mutations are present in 30–50% of non-small cell lung cancers and these mutations are associated with a favorable response to EGFR tyrosine kinase inhibitors [27]. NTRK1 encodes for TRKA, which belongs to the TRK family of proteins (composed by TRKA and its homologs TRKB and TRKC). Gene fusions involving NTRK act as oncogenic drivers of a broad diversity of tumors, and TRKs have become promising antitumor targets [28]. Finally, FBXO6 has been reported to play a role in cell cycle control, inactivating S-phase and spindle checkpoints, and might contribute to increase tumor cell resistance to certain anticancer drugs [29,30]. It will be important to study the functional consequences of these interactions in cancer development, progression and response to treatment. Altogether, our results provide a solid starting point for future studies on FASTK genes in cancer research and therapy.

## 4. Materials and Methods

### 4.1. TCGA Data Source Selection and Processing for Genetic Alterations Analysis

Somatic mutations and copy number alterations (CNAs) profiles of each of the FASTK genes were analysed using the TCGA Pan-Cancer dataset (32 studies and 10,967 tumor samples). Gene fusion predictions were also analyzed. We used the cBioPortal online tool (https://www.cbioportal.org, accessed on 10 July 2020) to analyze the TCGA Pan-Cancer dataset for mutations, CNAs, and gene fusions data [31,32]. The complete list of cancer types and information about normal tissue and primary tumor samples are provided in Table S3. Genomic alterations (i.e., missense, nonsense, frameshift and in-frame indels, splice-site, gene fusion) in FASTK family members are provided in Tables S1 and S2. Synonymous mutations were excluded from the analysis -as indicated by cBioPortal-with the exception of mutations localized in splice sites. The studied mutations are annotated according to the canonical UniProt transcript (https://www.uniprot.org, accessed on 10 July 2020) and protein domain definitions come from the PFAM database (http://pfam.xfam.org/, accessed on 10 July 2020). Lollipop visualizations were generated using the online tool MutationMapper (https://www.cbioportal.org/mutation_mapper, accessed on 15 July 2020). Patterns of gene alterations across samples in the Pan-Cancer dataset and summary of all relevant genomic alterations that exist in an individual tumor type were obtained using the OncoPrint tool in cBioPortal. The co-occurrence between mutated FASTKD1 and mutated FASTKD5 and mutations in other genes was analyzed using the Comparison/Survival tab (i.e., Genomic Alterations) available through cBioPortal. Samples were divided into groups with FASTKD1 mutation, FASTKD5 mutation, or not. The statistical analysis was performed using One-sided Fisher Exact Test as run by cBioPortal.

We used the Integrative Oncogenomics (IntOGen) online tool (https://www.intogen.org, accessed on 20 August 2021) [33] to identify driver mutations in FASTK genes. IntOGen incorporates seven state-of-the-art driver identification methods: dNdScv, cBaSe, OncodriveCLUSTL, HotMAPS3D, smregions, OncodriveFML and Mut-panning.

### 4.2. TCGA Data Source Selection and Processing for Expression Analysis

Expression profiles of the FASTK genes in TCGA were investigated using the UALCAN (http://ualcan.path.uab.edu/, accessed on 20 October 2020) [34], GEPIA2 (http://gepia2.cancer-pku.cn, accessed on 20 August 2021) [35], and cBioPortal (https://www.cbioportal.org, accessed on 15 July 2020) online tools. Publicly available TCGA RNA-seq data (“Level 3”) was used. We only considered tumor types for which we had ≥10 matched normal samples. The dataset contained 6903 and 709 samples for 16 cancer types and matched normal tissues, respectively (Table S3). The transcripts per million (TPM) were used as the measure of expression, because it has been suggested to be more comparable across samples than Fragments Per Kilobase of transcript per Million mapped reads (FPKM) and Reads Per Kilobase of transcript per Million mapped reads (RPKM) [36]. The cBioPortal analysis of mRNA expression uses RSEM batch normalized data (https://www.cbioportal.org, accessed on 15 July 2020). The statistical significance (*p*-values) of normal-vs-tumor differences in gene expression levels was estimated by Student’s *t*-test. Significantly different TPM values (*p* < 0.001) were highlighted (see Table S3).

Correlation between FASTKD3 expression and CNAs in LUSC, LUAD, ESCA, and BLCA was examined using the TCGA Pan-Cancer datasets through the Plots tab (i.e., mRNA-vs-CNA) in cBioPortal. Statistical analyses were performed using SPSS software version 24. The non-parametric Kruskal-Wallis H-test was used for testing differences in mRNA expression among three or more groups. For pairwise comparison the non-parametric Mann–Whitney U test was used.

### 4.3. Construction of the Protein-Protein Interaction Network of FASTK Proteins

For the PPI network analysis, we follow a similar approach with small modifications that was described in studying germline and somatic variants in DNA-Damage Response proteins [37]. Functional relationships among FASTK proteins were studied using PPI data from the BioGRID (https://thebiogrid.org/, accessed on 10 July 2020) [38], IntAct (https://www.ebi.ac.uk/intact/, accessed on 10 July 2020) [39] and STRING (https://string-db.org/, accessed on 10 July 2020) [40] databases. These repositories annotate PPIs from proteome-wide large-scale screenings and small-scale traditional studies. We assumed that by aggregating all known PPI experiments, one could be more confident of the interaction the more times a particular PPI occurred. The annotated human PPIs for the six FASTK proteins were aggregated, resulting in 185 proteins (nodes) and 278 interactions (edges). The topological parameters of the undirected FASTKs-PPI network were computed with the Cytoscape plugin NetworkAnalyzer (http://apps.cytoscape.org/apps/networkanalyzer, accessed on 12 July 2020) (Table S4). We also identified the most densely connected proteins or clusters in the FASTKs-PPI network using the MCODE algorithm as implemented in Metascape (http://metascape.org, accessed on 12 July 2020) [41]. Gene ontology and pathway enrichment analysis was done with Metascape.

## Figures and Tables

**Table 1:** List of cancer types.

Abbreviation	Cancer Type
ACC	Adrenocortical carcinoma
AML	Acute myeloid leukemia
BLCA	Bladder urotelial carcinoma
BRCA	Breast invasive carcinoma
CESC	Cervical squamous cell carcinoma and endocervical adenocarcinoma
CHOL	Cholangiocarcinoma
COAD	Colorectal adenocarcinoma
DLBC	Lymphoid Neoplasm Diffuse Large B-cell Lymphoma
ESCA	Esophageal carcinoma
GBM	Glioblastoma multiforme
HNSC	Head and neck squamous cell carcinoma
KICH	Kidney Chromophobe carcinoma
KIRC	Kidney renal clear cell carcinoma
KIRP	Kidney renal papillary cell carcinoma
LGG	Brain Lower Grade Glioma
LIHC	Liver hepatocellular carcinoma
LUAD	Lung adenocarcinoma
LUSC	Lung squamous cell carcinoma
MESO	Mesothelioma
OV	Ovarian serous cystadenocarcinoma
PAAD	Pancreas adenocarcinoma
PCPG	Pheochromocytoma and paraganglioma
PRAD	Prostate adenocarcinoma
READ	Rectal adenocarcinoma
SARC	Sarcoma
SKCM	Skin cutaneous melanoma
STAD	Stomach adenocarcinoma
TGCT	Testicular Germ Cell Tumors
THCA	Thyroid carcinoma
THYM	Thymoma
UCEC	Uterine Corpus Endometrial Carcinoma
UCS	Uterine Carcinosarcoma
UVM	Uveal Melanoma

**Table 2:** Pathway (REACTOME, KEEG, Canonical) and GO-term enrichment analysis (top three best *p*-value).

**Cluster 1**	**Log10 (*p*)**
(R-HSA-5205685) Pink/Parkin Mediated Mitophagy	−39.5
(R-HSA-5205647) Mitophagy	−37.1
(R-HSA-9663891) Selective autophagy	−29.7
**Cluster 2**	**Log10 (*p*)**
(hsa05200) Pathways in cancer	−7.2
(hsa04144) Endocytosis	−6.1
(M153) PID P75 NTR Pathway	−5.9
**Cluster 3**	**Log10 (*p*)**
(GO:0032543) Mitochondrial translation	−7.2
(GO:0140053) Mitochondrial gene expression	−6.9
(R-HSA-5693607) Processing of DNA double-strand break ends	−5.5

## Supplementary Materials

The following are available online at https://www.mdpi.com/article/10.3390/ijms222111337/s1, Table S1: Complete list of alterations of the FASTK gene family in cancer, Table S2: Complete list of the FASTK genes mutations and the types of cancer where they were found, Table S3: List of cancer types, number of tumor and normal tissue samples, and statistical significance in the gene expression changes, Table S4: Topological parameters of the PPI network, Figure S1: Co-occurrence mutation pattern of FASTKD1 and FASTKD5 in UCEC and SKCM, Figure S2: Correlation between FASTKD3 expression and CNAs in ESCA, LUSC, LUAD, and BLCA. Figure S3: Cumulative frequencies of genetic alterations of FASTK genes across cancers, Figure S4: Expression of FASTK genes across cancer tissues and matched normal tissues using GEPIA2 tool.

## Abbreviations

Catalogue Of Somatic Mutations In Cancer (COSMIC), clathrin heavy chain (CLTC), DD1 interacting factor-1 (DIF-1), epidermal growth factor receptor (EGFR), Fas-activated serine/threonine kinase (FASTK), FAST kinase Domains 1–5 (FASTKD1-5), FAST kinase-like protein, subdomain 1 (FAST_1), FAST kinase-like protein, subdomain 2 (FAST_2), F-box protein 6 (FBXO6), HECT and RLD domain containing E3 ubiquitin protein ligase 2 (HERC2), heterogeneous nuclear ribonucleoprotein A1 (HNRNPA1), heterogeneous nuclear ribonucleoprotein H2 (HNRNPH2), heat shock protein 90 alpha family class A member 1 (HSP90AA1), mitochondrial ribosomal protein L4 (MRPL4), mitochondrial ribosomal protein L58 (MRPL58), mitochondrially encoded ATP synthase 8/6 (MT-ATP8/6), mitochondrially encoded cytochrome c oxidase I (MT-CO1), mitochondrially encoded cytochrome c oxidase III (MT-CO3), mitochondrially encoded cytochrome b (MT-CYB), mitochondrially encoded NADH dehydrogenase 3 (MT-ND3), mitochondrially encoded NADH dehydrogenase 5 (MT-ND5), mitochondrially encoded NADH dehydrogenase 6 (MT-ND6), RNA binding motif protein X-linked (RBMX), nuclear receptor interacting factor-3 (NRIF3), neurotrophic receptor tyrosine kinase 1 (NTRK1), PHD finger protein 5A (PHF5A), TNF receptor associated factor 6 (TRAF6), pentatricopeptide repeat domain 1 (PTCD1), transforming growth factor beta regulator 4 (TBRG4), ubiquitin C (UBC), ubiquitin specific peptidase 7 (USP7), oxidative phosphorylation (OXPHOS), protein-protein interaction (PPI), The Cancer Genome Atlas (TCGA).
