# Supplementary material for: Systematic Analysis of FASTK Gene Family Alterations in Cancer
Source: Int J Mol Sci. 2021 Oct 20;22(21):11337. doi: 10.3390/ijms222111337 (PMC8583194; doi:10.3390/ijms222111337)
Supplement: Supplementary file 1 [file ijms-22-11337-s001.zip › Figure S1.pdf]

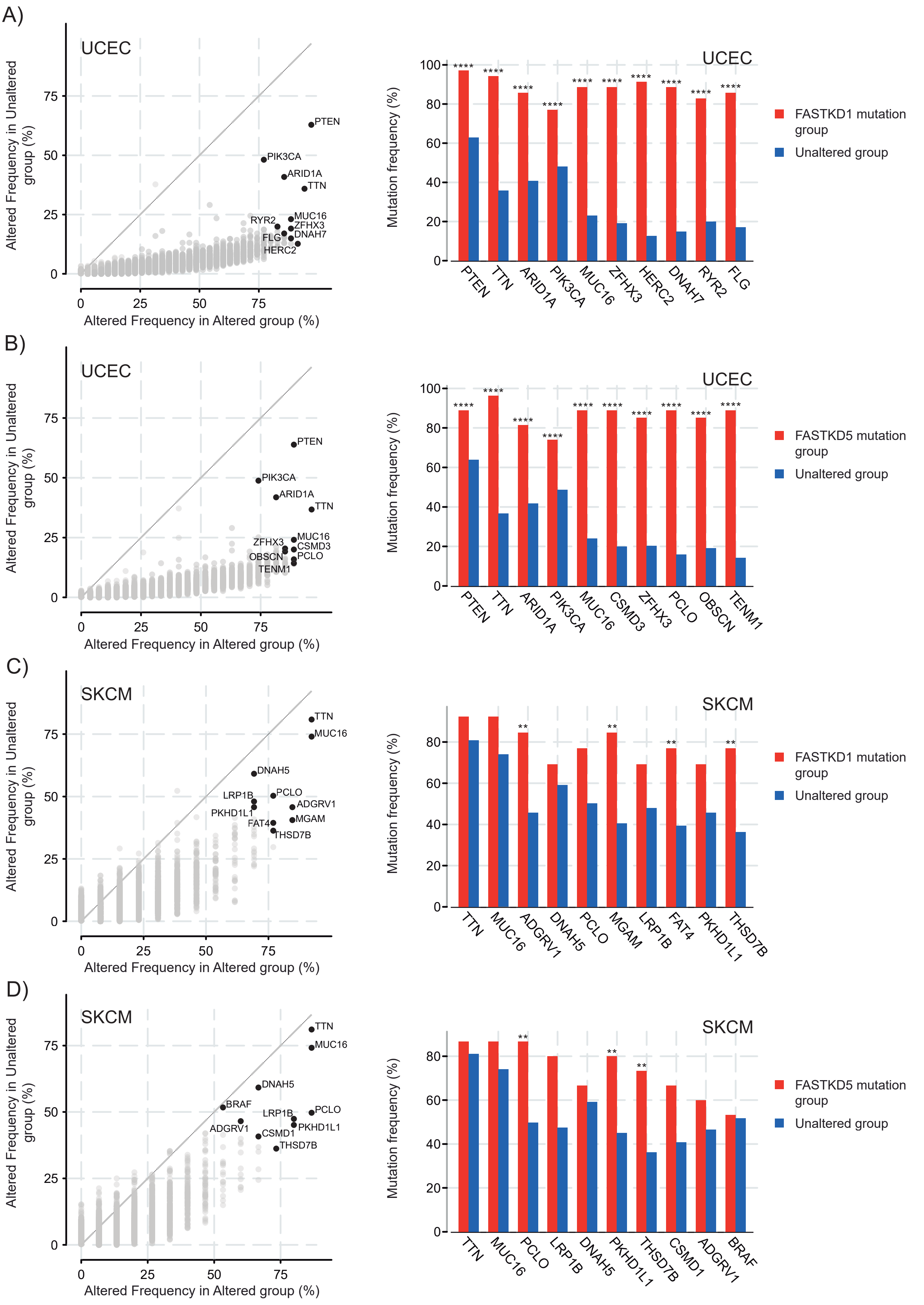

Figure S1: Co-occurrence mutation pattern of FASTKD1 (A,C) and FASTKD5 (B,D) and 10 genes with highest mutation frequency of each group in UCEC (A,B) and SKCM (C,D). ** p<0.01; **** P<0.0001
