# Supplementary material for: Systematic Analysis of FASTK Gene Family Alterations in Cancer
Source: Int J Mol Sci. 2021 Oct 20;22(21):11337. doi: 10.3390/ijms222111337 (PMC8583194; doi:10.3390/ijms222111337)
Supplement: Supplementary file 1 [file ijms-22-11337-s001.zip › Figure S2.pdf]

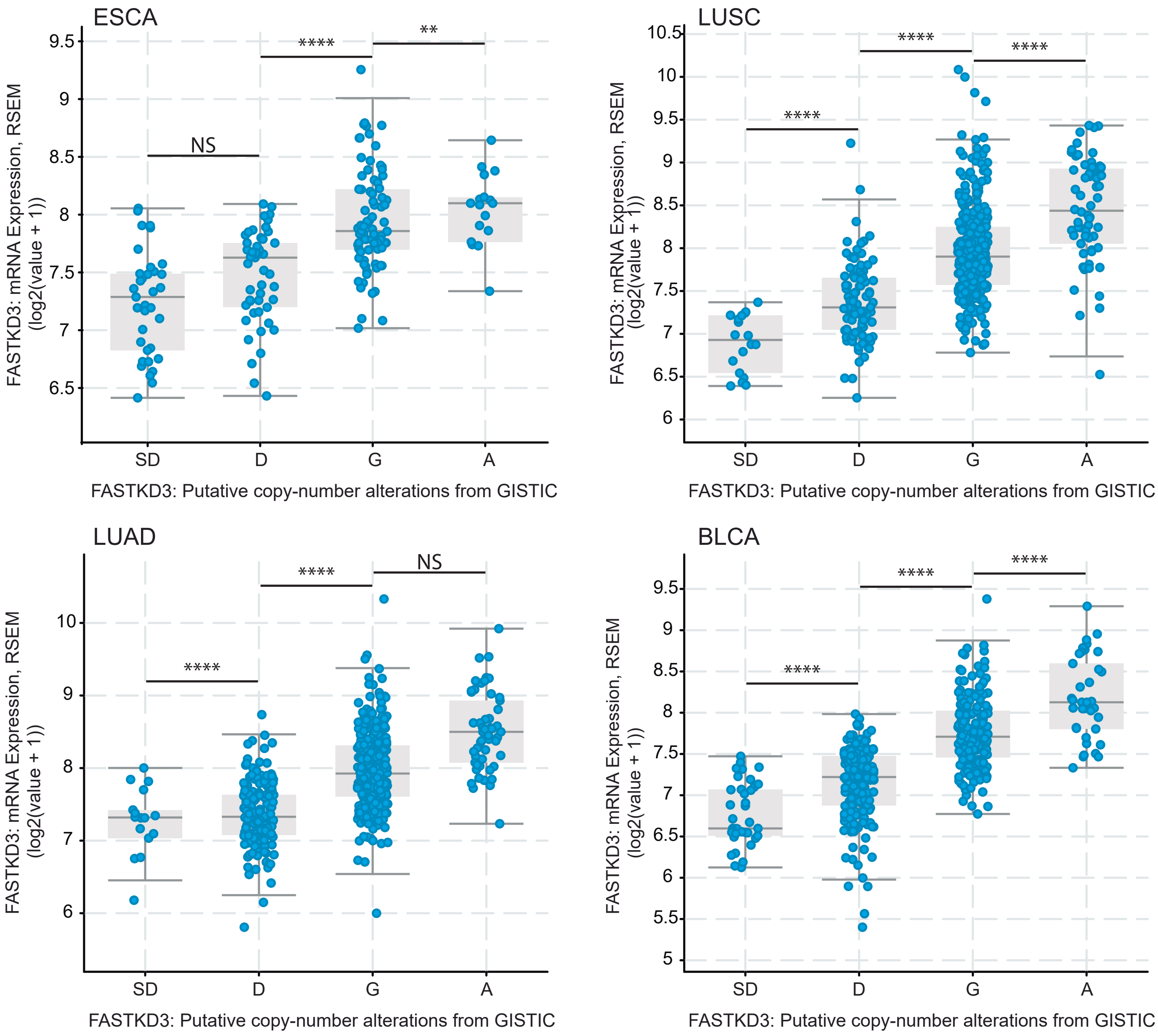

Figure S2. Correlation between FASTKD3 expression and CNAs in ESCA, LUSC, LUAD, and BLCA. DD, deep deletion; SD, shallow deletion; D, diploid; G, gain; A, amplification. NS, not significant; ** p<0.01; **** p<0.0001.
