# Supplementary material for: Systematic Analysis of FASTK Gene Family Alterations in Cancer
Source: Int J Mol Sci. 2021 Oct 20;22(21):11337. doi: 10.3390/ijms222111337 (PMC8583194; doi:10.3390/ijms222111337)
Supplement: Supplementary file 1 [file ijms-22-11337-s001.zip › Figure S3.pdf]

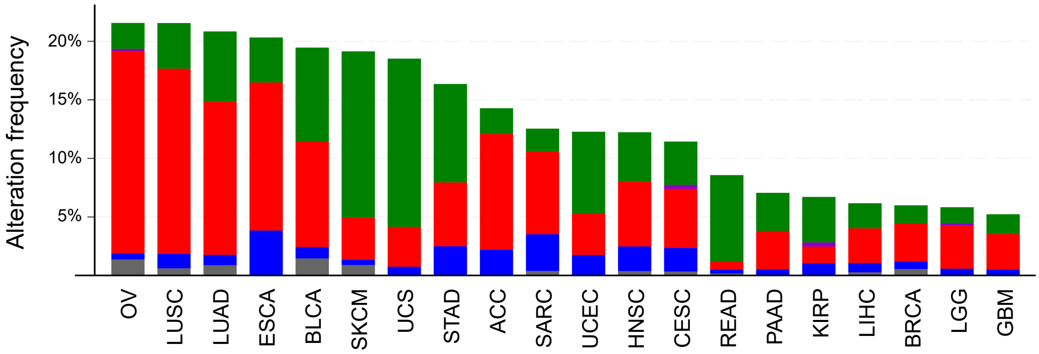

Figure S3. Cumulative frequencies of genetic alterations of FASTK genes across the 20 cancer types harboring the highest alteration rates.
