# Supplementary material for: Systematic Analysis of FASTK Gene Family Alterations in Cancer
Source: Int J Mol Sci. 2021 Oct 20;22(21):11337. doi: 10.3390/ijms222111337 (PMC8583194; doi:10.3390/ijms222111337)
Supplement: Supplementary file 1 [file ijms-22-11337-s001.zip › Figure S4.pdf]

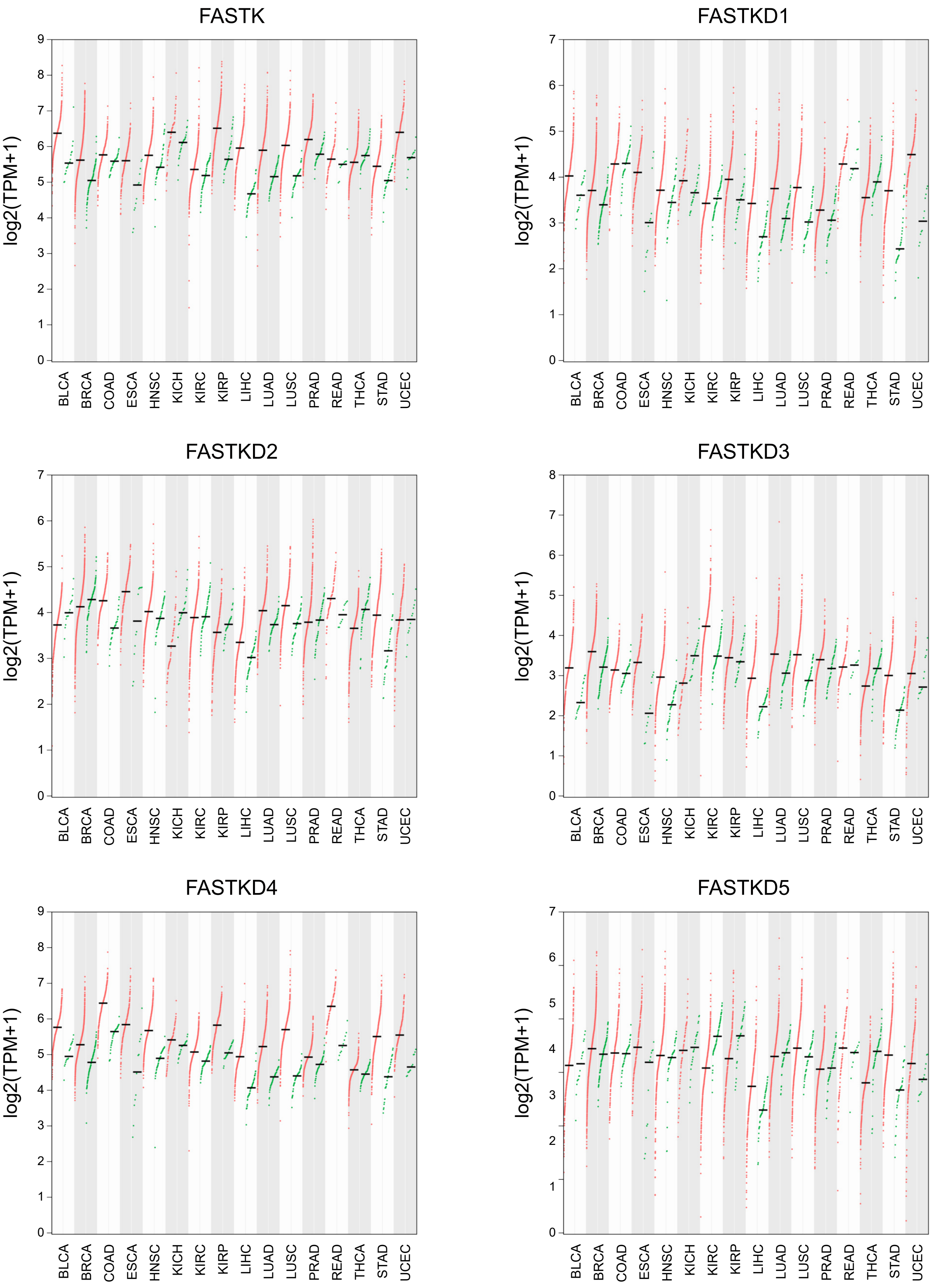

Figure S4. Expression of FASTK genes across 16 cancer types (red) and matched normal tissues (green) using GEPIA tool
