# Supplementary material for: Systematic Analysis of FASTK Gene Family Alterations in Cancer
Source: Int J Mol Sci. 2021 Oct 20;22(21):11337. doi: 10.3390/ijms222111337 (PMC8583194; doi:10.3390/ijms222111337)
Supplement: Supplementary file 1 [file ijms-22-11337-s001.zip › Table S3.pdf]

Table S3. List of cancer types, number of tumor and normal tissue samples, and statistical significance in the gene expression changes.

Abbreviation	Cancer type	Tumor	Normal	Statistical significance (gene expression)					
				FASTK	FASTKD1	FASTKD2	FASTKD3	TBRG4	FASTKD5
ACC	Adrenocortical carcinoma	79	0	n.a.	n.a.	n.a.	n.a.	n.a.	n.a.
AML	Acute myeloid leukemia	173	0	n.a.	n.a.	n.a.	n.a.	n.a.	n.a.
BLCA	Bladder urotelial carcinoma	408	19	**3.8×10^{-12}**	5.3×10^{-02}	**7.9×10^{-04}**	**1.7×10^{-11}**	**1.9×10^{-12}**	6.8×10^{-01}
BRCA	Breast invasive carcinoma	1097	114	**1.6×10^{-12}**	**1.9×10^{-08}**	**1.5×10^{-10}**	**1.1×10^{-16}**	**$< 1 \times 10^{-12}$**	**1.9×10^{-05}**
CESC	Cervical squamous cell carcinoma and endocervical adenocarcinoma	305	3	n.a.	n.a.	n.a.	n.a.	n.a.	n.a.
CHOL	Cholangiocarcinoma	36	9	n.a.	n.a.	n.a.	n.a.	n.a.	n.a.
COAD	Colorectal adenocarcinoma	286	41	**1.4×10^{-03}**	**6.5×10^{-03}**	**1.6×10^{-12}**	**4.8×10^{-03}**	**$< 1 \times 10^{-12}$**	1.2×10^{-02}
DLBC	Lymphoid Neoplasm Diffuse Large B-cell Lymphoma	48	0	n.a.	n.a.	n.a.	n.a.	n.a.	n.a.
ESCA	Esophageal carcinoma	184	11	**4.3×10^{-05}**	**4.7×10^{-06}**	**9.3×10^{-03}**	**7.7×10^{-08}**	**5.7×10^{-10}**	**2×10^{-03}**
GBM	Glioblastoma multiforme	156	5	n.a.	n.a.	n.a.	n.a.	n.a.	n.a.
HNSC	Head and neck squamous cell carcinoma	520	44	**1×10^{-04}**	3.4×10^{-02}	**1.2×10^{-03}**	**$<1 \times 10^{-12}$**	**$<1 \times 10^{-12}$**	**1.4×10^{-03}**
KICH	Kidney Chromophobe carcinoma	67	25	**8.5×10^{-07}**	**4.6×10^{-04}**	**3.9×10^{-08}**	**5.8×10^{-04}**	**4.9×10^{-08}**	2.6×10^{-01}
KIRC	Kidney renal clear cell carcinoma	533	72	**1.2×10^{-03}**	**1.7×10^{-12}**	1.7×10^{-02}	5.5×10^{-01}	**7.5×10^{-09}**	**$<1 \times 10^{-12}$**
KIRP	Kidney renal papillary cell carcinoma	290	32	**1.6×10^{-12}**	9.7×10^{-01}	1.1×10^{-01}	5.8×10^{-01}	**1.6×10^{-12}**	**1.7×10^{-04}**
LGG	Brain Lower Grade Glioma	513	0	n.a.	n.a.	n.a.	n.a.	n.a.	n.a.
LIHC	Liver hepatocellular carcinoma	371	50	**1.6×10^{-12}**	**1.6×10^{-12}**	**1.6×10^{-12}**	**$<1 \times 10^{-12}$**	**1.6×10^{-12}**	**1.6×10^{-12}**
LUAD	Lung adenocarcinoma	515	59	**1.6×10^{-12}**	**1.6×10^{-12}**	**3.3×10^{-16}**	**1.6×10^{-12}**	**$<1 \times 10^{-12}$**	1.1×10^{-01}
LUSC	Lung squamous cell carcinoma	503	52	**$<1 \times 10^{-12}$**	**$<1 \times 10^{-12}$**	**2.1×10^{-13}**	**$<1 \times 10^{-12}$**	**$<1 \times 10^{-12}$**	**3×10^{-12}**
MESO	Mesothelioma	87	0	n.a.	n.a.	n.a.	n.a.	n.a.	n.a.
OV	Ovarian serous cystadenocarcinoma	305	0	n.a.	n.a.	n.a.	n.a.	n.a.	n.a.
PAAD	Pancreas adenocarcinoma	178	4	n.a.	n.a.	n.a.	n.a.	n.a.	n.a.
PCPG	Pheochromocytoma and paraganglioma	179	3	n.a.	n.a.	n.a.	n.a.	n.a.	n.a.
PRAD	Prostate adenocarcinoma	497	52	**1.6×10^{-12}**	6.4×10^{-01}	**1.2×10^{-03}**	**2.3×10^{-08}**	**5.7×10^{-09}**	6.5×10^{-01}
READ	Rectal adenocarcinoma	166	10	**1.5×10^{-05}**	4.3×10^{-02}	**7.8×10^{-03}**	7.9×10^{-01}	**1.6×10^{-12}**	8.4×10^{-02}
SARC	Sarcoma	260	2	n.a.	n.a.	n.a.	n.a.	n.a.	n.a.
SKCM	Skin cutaneous melanoma	472	1	n.a.	n.a.	n.a.	n.a.	n.a.	n.a.
STAD	Stomach adenocarcinoma	415	34	**2×10^{-04}**	**7.2×10^{-14}**	**1×10^{-12}**	**$<1 \times 10^{-12}$**	**1.6×10^{-12}**	**$<1 \times 10^{-12}$**
TGCT	Testicular Germ Cell Tumors	150	0	n.a.	n.a.	n.a.	n.a.	n.a.	n.a.
THCA	Thyroid carcinoma	505	59	**2.3×10^{-03}**	**1.6×10^{-12}**	**1.6×10^{-12}**	**6.9×10^{-10}**	**6.4×10^{-07}**	**1.6×10^{-12}**
THYM	Thymoma	120	2	n.a.	n.a.	n.a.	n.a.	n.a.	n.a.
UCEC	Uterine Corpus Endometrial Carcinoma	546	35	**3.2×10^{-09}**	**4.9×10^{-11}**	**7.6×10^{-05}**	2.9×10^{-01}	**1.6×10^{-12}**	**1.1×10^{-10}**
UCS	Uterine Carcinosarcoma	57	0	n.a.	n.a.	n.a.	n.a.	n.a.	n.a.
UVM	Uveal Melanoma	80	0	n.a.	n.a.	n.a.	n.a.	n.a.	n.a.

Statistically significant p-values are in bold.
