# Supplementary material for: Systematic Analysis of FASTK Gene Family Alterations in Cancer
Source: Int J Mol Sci. 2021 Oct 20;22(21):11337. doi: 10.3390/ijms222111337 (PMC8583194; doi:10.3390/ijms222111337)
Supplement: Supplementary file 1 [file ijms-22-11337-s001.zip › Table S4.pdf]

Table S4. Topological parameters of the PPI network

Parameters	Mean
AverageShortestPathLength	3.763396005
BetweennessCentrality	0.015100522
ClosenessCentrality	0.273714652
ClusteringCoefficient	0.085340065
Degree	3.005405405
Eccentricity	5.805405405
NeighborhoodConnectivity	40.24580169
Radiality	0.605229143
Stress	5100.259459
TopologicalCoefficient	0.132442709
